# Numerical evaluation of internal femur osteosynthesis based on a biomechanical model of the loading in the proximal equine hindlimb

**DOI:** 10.1186/s12917-024-04044-5

**Published:** 2024-05-10

**Authors:** Jan J. Lang, Xinhao Li, Carina M. Micheler, Nikolas J. Wilhelm, Fritz Seidl, Benedikt J. Schwaiger, Dirk Barnewitz, Ruediger von Eisenhart-Rothe, Christian U. Grosse, Rainer Burgkart

**Affiliations:** 1 Department of Orthopedics and Sports Orthopedics, Klinikum rechts der Isar, TUM School of Medicine, Technical University of Munich, Munich, Germany; 2 Chair of Non-destructive Testing, TUM School of Engineering and Design, Technical University of Munich, Munich, Germany; 3 Institute for Machine Tools and Industrial Management, TUM School of Engineering and Design, Technical University of Munich, Munich, Germany; 4 Munich Institute of Robotics and Machine Intelligence, Department of Electrical and Computer Engineering, Technical University of Munich, Munich, Germany; 5 Department of Diagnostic and Interventional Neuroradiology, TUM School of Medicine, Technical University of Munich, Munich, Germany; 6 Equine Clinic of the Research Centre for Medical Technology and Biotechnology, Bad Langensalza, Germany

**Keywords:** Musculoskeletal model, FEA, Intramedullary nail, Horse, Fracture

## Abstract

Femoral fractures are often considered lethal for adult horses because femur osteosynthesis is still a surgical challenge. For equine femur osteosynthesis, primary stability is essential, but the detailed physiological forces occurring in the hindlimb are largely unknown. The objective of this study was to create a numerical testing environment to evaluate equine femur osteosynthesis based on physiological conditions. The study was designed as a finite element analysis (FEA) of the femur using a musculoskeletal model of the loading situation in stance. Relevant forces were determined in the musculoskeletal model via optimization. The treatment of four different fracture types with an intramedullary nail was investigated in FEA with loading conditions derived from the model. The analyzed diaphyseal fracture types were a transverse (TR) fracture, two oblique fractures in different orientations (OB-ML: medial-lateral and OB-AP: anterior-posterior) and a ”gap” fracture (GAP) without contact between the fragments. For the native femur, the most relevant areas of increased stress were located distally to the femoral head and proximally to the caudal side of the condyles. For all fracture types, the highest stresses in the implant material were present in the fracture-adjacent screws. Maximum compressive (-348 MPa) and tensile stress (197 MPa) were found for the GAP fracture, but material strength was not exceeded. The mathematical model was able to predict a load distribution in the femur of the standing horse and was used to assess the performance of internal fixation devices via FEA. The analyzed intramedullary nail and screws showed sufficient stability for all fracture types.

**Supplementary Information:**

The online version contains supplementary material available at 10.1186/s12917-024-04044-5.

## Introduction

Osteosynthesis for femoral fractures in adult horses requires demanding surgery and is often considered as non-promising [1], which is accompanied with an emotional and economical loss for the horses’ owners. There are several factors that make the procedure difficult for this type of bone. The horses’ femur is covered with a considerable mass of muscle tissue, which makes surgical access challenging. The time of surgery is limited due to increasing mortality with increased time on anesthesia [2]. In addition, complete postoperative unloading of the fixed limb is hard to achieve and often not tolerated by the animal [3]. Consequently, horses must stand up during the recovery period from anesthesia. This can lead to uncoordinated movements and insufficient or disadvantageous attempts to stand up, resulting in peak loads for the osteosynthesis [4]. Consequently, primary stability is extremely important for this type of osteosynthesis. In order to address these challenges, an internal fixation for the equine femur was developed in cooperation with the Equine Clinic of the Research Centre for Medical Technology and Biotechnology GmbH (Bad Langensalza, GER) and Königsee GmbH (Allendorf, GER). The newly developed intramedullary nail is designed to withstand high loads and to create a high primary stability. For the femur, the primary stability of this implant and its further improvement via fracture-adjacent reinforcement was already investigated by Lang et al. in biomechanical testing [5].

Nevertheless, the concept of using intramedullary nailing for the treatment of equine femur fractures is not new. Several studies tested and evaluated this method on different equine long bones [6–11]. Radcliffe et al. performed a biomechanical comparison on intermedullary nailing against double plating of fractures [10]. In vivo evaluation of intramedullary fixation of the femur was performed by McClure et al. [11]. The femora of six young horses (149–207 kg) were treated with an interlocking nail and the healing rate was 100%. But the dimensions of the used implants in both studies are comparable to the intramedullary nails from human medicine. Consequently, only the application for foals and yearlings was promoted. In contrast, the newly developed implant used in this study is designed for the application in adults. Nevertheless, the bone seems to be prone to high loads regarding the femur dimensions, which is challenging for all kinds of osteosynthesis implants. But so far, there is insufficient knowledge about how exactly these loads are acting on the equine femur. Literature lacks information about the stress distribution in this bone in different motion conditions. In vivo strain gauges have been attached to tibia and metatarsus [12], but not the femur. Frazer et al. did two *in silico* studies on the influence of a subchondreal bone cyst on the stresses in the femoral condyles [13, 14]. Due to the lack of information, they had to estimate the forces for the femur as well as the muscle force acting on the patella. But getting to know the stresses and strains more precisely would be advantageous for optimizing the positioning, the fixation and the design of the intramedullary nail. For areas of high stress, the placement of fixation screws should be avoided. Weakening the bone in these areas increases the chance of overloading the remaining bone and may lead to failure of the osteosynthesis. By incorporating this information into a model, it becomes possible to analyze various conditions for osteosynthesis, enabling their evaluation through comparative parameter studies, similar to the approach used in human subjects [15–17].

For this reason, we created a model for the physiological loading conditions while standing for the equine femur und used these results for the numerical evaluation of internal osteosynthesis on different fracture types.

## Methods

**Fig. 1:**
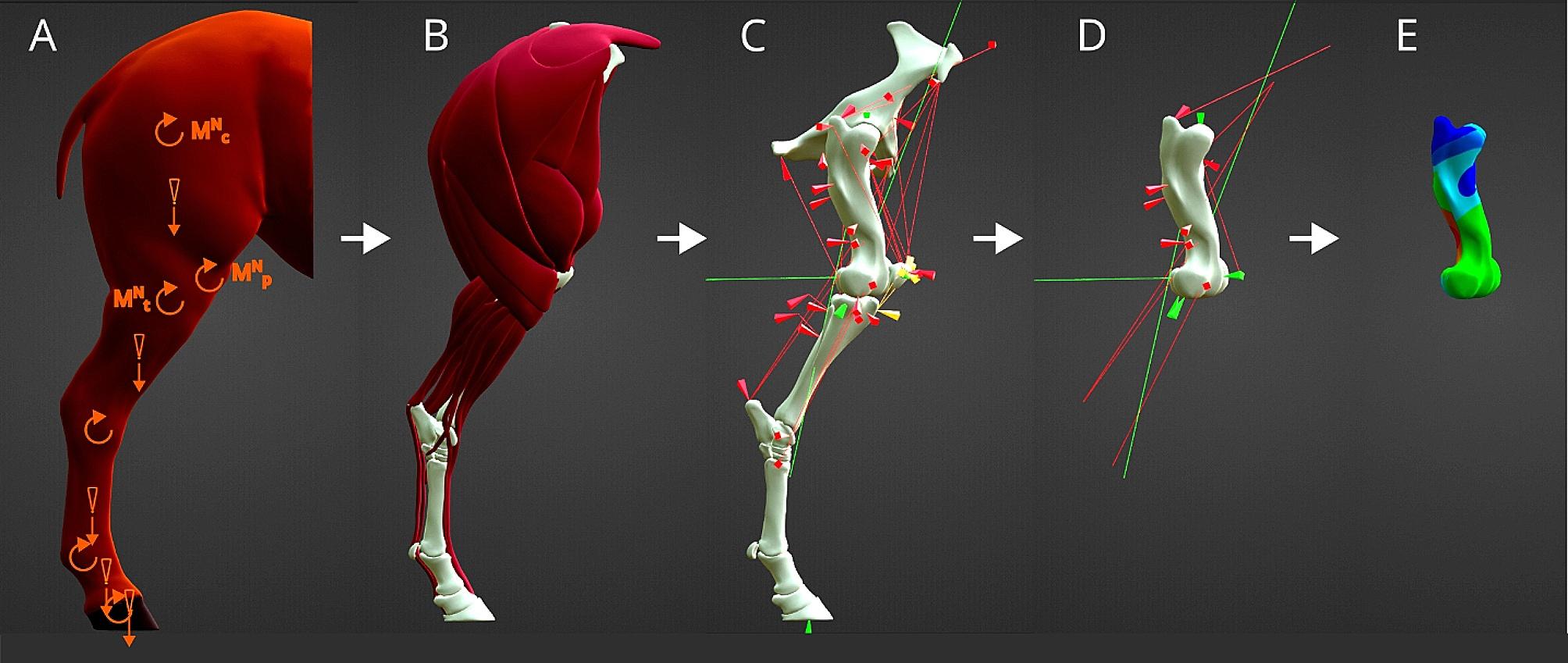
Schematic visualization of the procedure from model to FEA. (**A**) from the basic standing anatomy and joint angles, the segmental loads (orange straight arrows) and ground reaction forces are used to determine the intersegmental loads and net joint moments (orange curved arrows, top three are coxofemoral ${\overrightarrow{M}}_{c}^{N}$, femorotibial ${\overrightarrow{M}}_{t}^{N}$ and femoropatellar ${\overrightarrow{M}}_{p}^{N}$; (**B**) All muscles details are derived from literature and (**C**) attachments (cones) and direction (lines) are transferred onto the bone anatomy. All needed parts, muscles (red), ligaments (yellow) and contact forces (green) are combined to be represented in one coordinate system. (**D**) With optimization, the forces of the muscles and ligaments are determined and contact forces are calculated. Only relevant forces are included for the femur and (**E**) transferred into a Finite Element Analysis.

The following paragraph provides an overview on the procedure for this study. A mathematical model for the muscles acting in the proximal hindlimb while standing is developed. A similar approach was performed by Pollock et al. on the front limb [18]. All acting muscles on the femur and the surrounding bones are defined with their origin, insertion and acting direction. With a mathematical optimization approach, the acting muscle forces, which counteract the net joint moments caused by intersegmental loads, are identified. These acting muscles forces are used for a Finite Element Analysis (FEA) on the equine femur to evaluate areas of high stress and to interpret this information in relation to the positioning of the implant. The procedure is visualized in Fig. 1. Furthermore, the influence of the fracture type on the stress distribution in the bone and the implant parts is analyzed in the FEA model.

## Musculoskeletal model

### Bone anatomy

In order to obtain insights on the femoral loading during standing, we focused on muscles that either produce a moment for the coxofemoral, femorotibial and femoropatellar joint. Spatial information about the attachment sites and the effective orientation were combined in a 3D model. The model was built in the software *blender* (Version 3.01, Blender Foundation, Amsterdam, NL) based on a commercially available horse skeleton in standing position (Sketchfab Inc., New York, USA) with appropriate proportions and joint angles. In order to have a matching bone with precise material distribution for the numerical model, one cadaveric femur specimen was chosen. The donor animal was an 18-year-old Haflinger gelding with a wither height of 148 cm, which died unrelated to this study and of which only the femur was obtained. The weight of the animal was unknown. In a study of Hois, the wither height and weight of Haflingers were estimated with modified Janoschek functions from foal data [19]. The resulting average wither height of adults was 149 cm, which matches the wither height of our donor animal. For this reason, the resulting weight of 514 kg was chosen as estimation of the weight of the donor animal for the calculation in the model. This weight was used for determination of ground reaction loads and intersegmental loads. To obtain a distinction of cortical and cancellous bone for the FEA, the femur specimen was scanned with a standard computed tomography device (IQon – Spectral CT, Philips, Amsterdam, NL). The resolution of the scan resulted in a voxel size of 0.43 × 0.43 × 0.67 mm^3^. The segmentation was performed with a tool in the software *Dragonfly* (Version 2022, ORS Inc., Montreal, CA), which uses predefined Neural Networks and is capable of advanced distinction between cancellous and cortical bone for the whole femur. The exported mesh was repaired and smoothed in *Geomagic Wrap* (Version 2017, 3D Systems, Inc., Rock Hill, USA).

### Intersegmental loading

For the analysis of the loading in the hindlimb, several important points have to be defined for the model. The center of rotation for the hip was the center of the femoral head, which was found with sphere approximation. The simplified center of rotation for the femorotibial joint was the midpoint between the lateral and medial epicondyles of the femur [20] For the patellofemoral joint, the center of rotation was the contact point between patella and femur. The center of the joint is very complex, but for standing, it can be simplified by identifying it as a contact point. The coordinates from the model in *blender* were exported via *python* script. The preprocessing of the coordinate data, the creation of the object function as well as the optimization approach was done in *Matlab* (Version 2022b, The Mathworks Inc., Natick, USA). In order to calculate the forces that the muscles have to provide for standing, the intersegmental loads have to be determined. The intersegmental loads are the forces and moments at the joints that are caused by external forces. In case of standing, these are only constituted by gravitational forces caused by the weight of the main body and the segments of the limb. The resulting moments - also called net joint moments - have to be counteracted by the muscular forces to achieve a stable standing. Using the mass of the lower limb segments and ground reaction loads, intersegmental loads at the hip and stifle joint were calculated. In terms of ground reaction loads, it is known from the literature that each hindlimb bears about 20% of the entire weight at the center of the hoof when the horse is standing without without locking of the joint [21]. Symmetrical load bearing between the hind limbs was assumed. Following the procedure of Nauwelaerts et al., the hindlimbwas divided into five subunits (thigh, curs, metatarsus, hind pastern and hind hoof) and these were assigned with corresponding weights and centers of mass [22]. From the 3D model and the resulting intersegmental forces based on the ground reaction force, the net moments at the joints can be calculated. These must be compensated by the muscles to achieve a total moment of zero for the joints in the standing horse. The resulting values for the ground reaction force, the segmental loads and the net joint moments as well as their origins can be observed in Online Resource 1. The absolute values for the net moments are ${M}_{c}^{N}=104 Nm$ for the coxofemoral joint and${M}_{t}^{N}=109 Nm$ for the femorotibial joint. For the femoropatellar joint, a net moment ${M}_{p}^{N}$of zero was defined, as the patella has almost no gravitational influence.

### Muscle direction and attachment

**Fig. 2:**
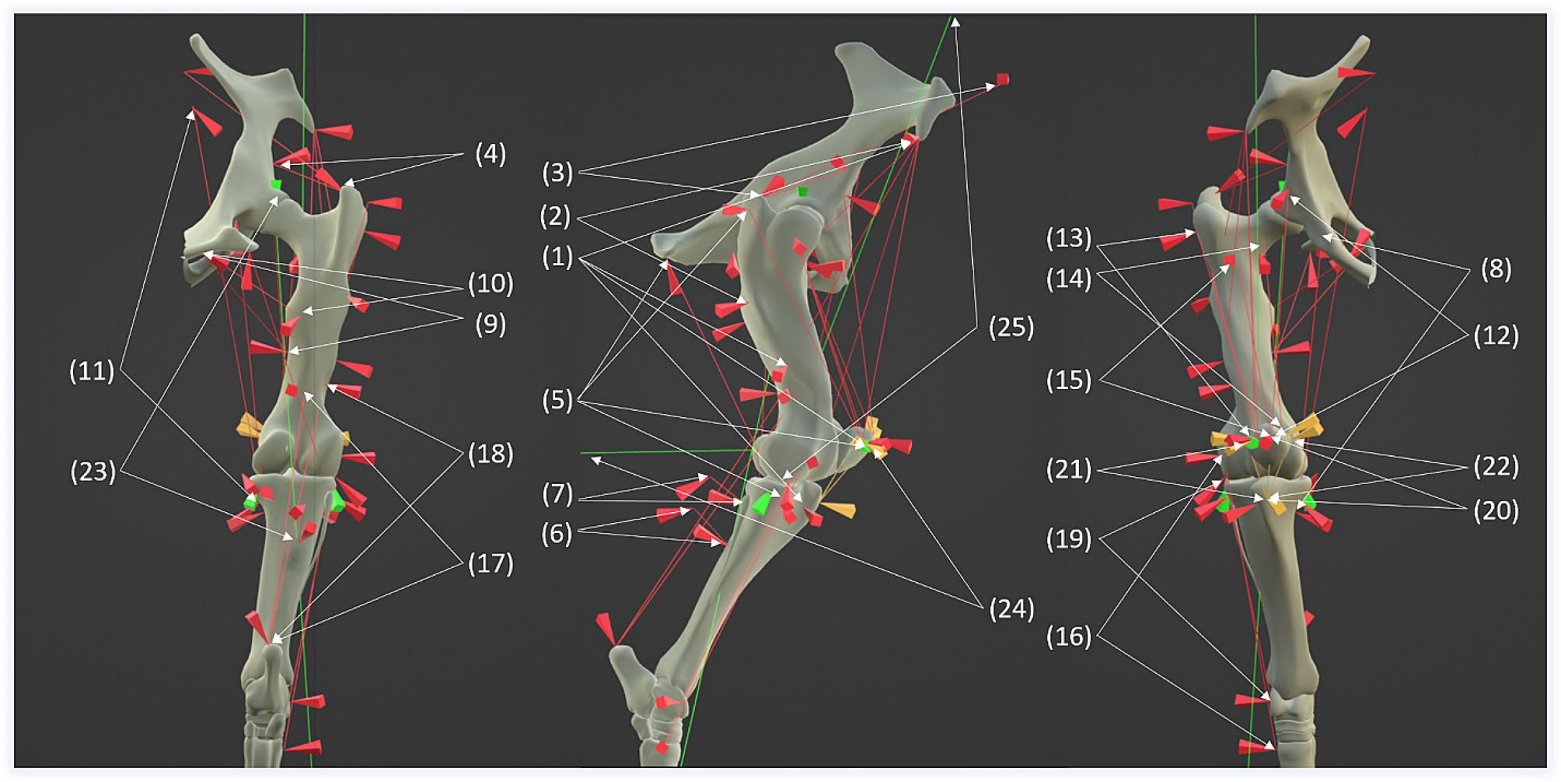
Representation of muscles, ligaments, and contact force. White arrows show origin and insertion of muscles (red), ligaments (yellow) and contact points for contact forces (green) in the joints (1) M. tensor fasciae latae (2) M. gluteus superficialis (3) M. gluteus medius (4) M. gluteus profundus (5) M. biceps femoris (6) M. semitendinosus (7) M. semimembranosus (8) M. gracilis (9) M. adductor (10) M. pectineus (11) M. sartorius (12) M. rectus femoris (13) M. vastus lateralis (14) M. vastus medialis (15) M. vastus intermedius (16) M. peroneus tertius (17) M. gastrocnemius (18) M. flexor digitalis superficialis (19) M. extensor digitalis longus (20) Lig. patellae intermediate (21) Lig. patellae lateralis (22) Lig. patellae medialis (23) coxofemoral contact force (24) femoropatellar contact force (25) femorotibial contact force.

The insertion and origin of muscles crossing the above-mentioned three joints were incorporated in the hind limb model (Fig. 2).

The locations of these muscle attachment sites were taken from the literature [23–26]. Muscle origin and insertion locations are specified in Online Resource 2 for the approach described here. For the mathematical analysis, the attachment site was simplified as a single point. This point was set in the center of the determined muscle attachment area. For the *m. tensor fascia latae*, the insertion area is widely spread along the femur axis. To account for this, the insertion area was divided and three points are used for approximation. One point was set on the femur, one was set on the patella and one was set on the tibia. The *m. gastrocnemius* is divided into a medial and lateral part with their origin located proximally to each condyle of the femur. These attachment areas were combined into a centrally located point for the musculoskeletal model. In general, the direction between the insertion and origin point was chosen as line of action for the muscle. For the *m. biceps femoris*, this approach was not suitable. *M. biceps femoris* is a long and strong muscle which is divided into three heads. For the model, the middle and caudal heads can be merged into one point. But due to the muscle curvature, the direction for the resulting muscle force of the *m. biceps femoris* was chosen as the direction of the muscle close to its insertion area instead of the direct link between origin and insertion. The same was done for the *m. semitendinosus* and *semimembranosus*. The ligaments that are connected to the patella form a complex structure. For the musculoskeletal model, only the patellar ligaments (*Lig. patellae intermediale, lig. patellae laterale, lig. patellae medialis*) responsible for the transmission of forces to the tibia were considered. A similar approach was taken by Fraser et al. for the numerical simulation of the equine knee [14]. These ligaments are responsible for redirecting the force of the *m. quadriceps* to the tibia and are therefore very important for the model. The origins for these ligaments are placed on the patella, while their insertion is at the tuberosity of tibia. The particular structure of the equine stifle joint ensures that the patellar ligaments run in different orientations despite having the same insertion and original sites. This was considered in the model. The coordinates of origin, insertion and direction of the muscles and ligaments used for the model can be found in Online Resource 3.

### Optimization

The coordinates for the muscle and ligament attachments, the centers of rotation, the centers of mass as well as the muscle direction were exported from the 3D model. For every joint, the corresponding muscles were determined due to their attachments and lines of action. The orientation vectors of the forces ${\overrightarrow{r}}_{i}$ were normalized and transferred to a linear system of equations together with the distance of the attachment point from the center of rotation ${ \overrightarrow{\tau }}_{i}$ as vector products. The resulting system of equations can be given by the following Eq. (1), with ${\overrightarrow{X}}^{F}$ as the force factor for all muscles.

1 $$\left(\begin{array}{c} {\overrightarrow{r}}_{i}\times { \overrightarrow{\tau }}_{i,c}\\ {\overrightarrow{r}}_{i}\times { \overrightarrow{\tau }}_{i,t}\\ {\overrightarrow{r}}_{i}\times { \overrightarrow{\tau }}_{i,p}\end{array}\right)\bullet {\overrightarrow{X}}^{F}=\left(\begin{array}{c}{\overrightarrow{M}}_{c}^{N}\\ {\overrightarrow{M}}_{t}^{N}\\ {\overrightarrow{M}}_{p}^{N}\end{array}\right)$$

The number of muscles (and ligaments) *i* exceeds the number of equations in the system. Consequently, no unique solution for ${\overrightarrow{X}}^{F}$ can be found. In order to find a suitable solution, an optimization method incorporating additional conditions is used. These conditions refer to an efficient distribution of the muscle forces [18]. The optimization approach minimizes the following Eq. (2):

2 $$\text{min}U= \sum _{i=1}{\left(\frac{{\overrightarrow{X}}_{i}^{F}}{{PCSA}_{i}}\right)}^{3}$$

${PCSA}_{i}$ is the physiological cross-sectional area and ${\overrightarrow{X}}_{i}^{F}$ is the corresponding force factor of the $i$*-*th muscle. This scalar objective function was set up in *Matlab* using the *fmincon* solver from the objective function toolbox. Additionally, conditions were included. The obtained forces were supposed to be between a maximum force, which corresponds to the maximum isometric force of the muscles provided by Payne et al. [25], and a minimum force. The minimum force is 1% of the maximum isometric force, which corresponds to the muscle resting tonus [27, 28]. Only for the m. superficial digital flexor, a minimum of 90% of the ground reaction force was used, which was taken from the approximation of Schuurmann et al. to incorporate the stabilizing moment of the hook joint [29]. The joint contact forces were calculated as combination of intersegmental forces and the forces created by the muscles acting across corresponding joint (3).

3 $${F}_{{contact}}= \sum {F}_{segmental}+\sum {F}_{muscles}$$

## Numerical analysis

### Osteosynthesis setup and fracture types

**Fig. 3:**
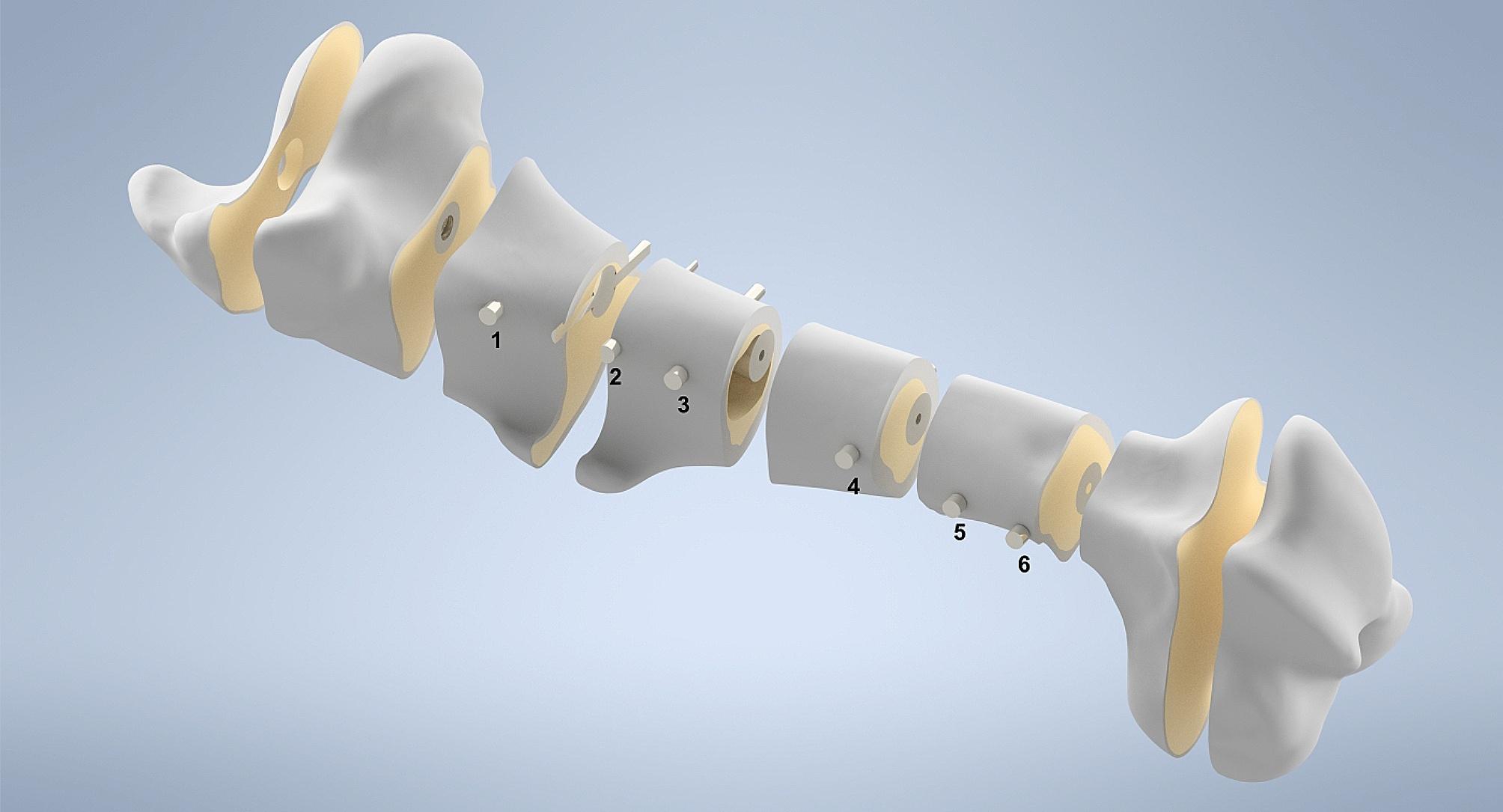
Sectional view of the equine femur Graphic illustration of the distinction between cortical (gray) and cancellous bone (yellow) and the inserted intramedullary nail including screws (metallic). The numbers shown are used for the position designation of the fixation screws

The 3D models of the cortical and cancellous bone were imported into the CAD (computer-aided design) software *SolidWorks* (Version 2022, Daussault Systems, Vélizy-Villacoublay, FR). In this environment, the intramedullary nail and the fixation screws were digitally incorporated into the model. Boolean operations were used to create the bone cavities for the implanted parts. Figure 3 visualizes how the implant and the screws are incorporated into the bone as well as the local distinction between cortical and cancellous bone.

**Fig. 4:**
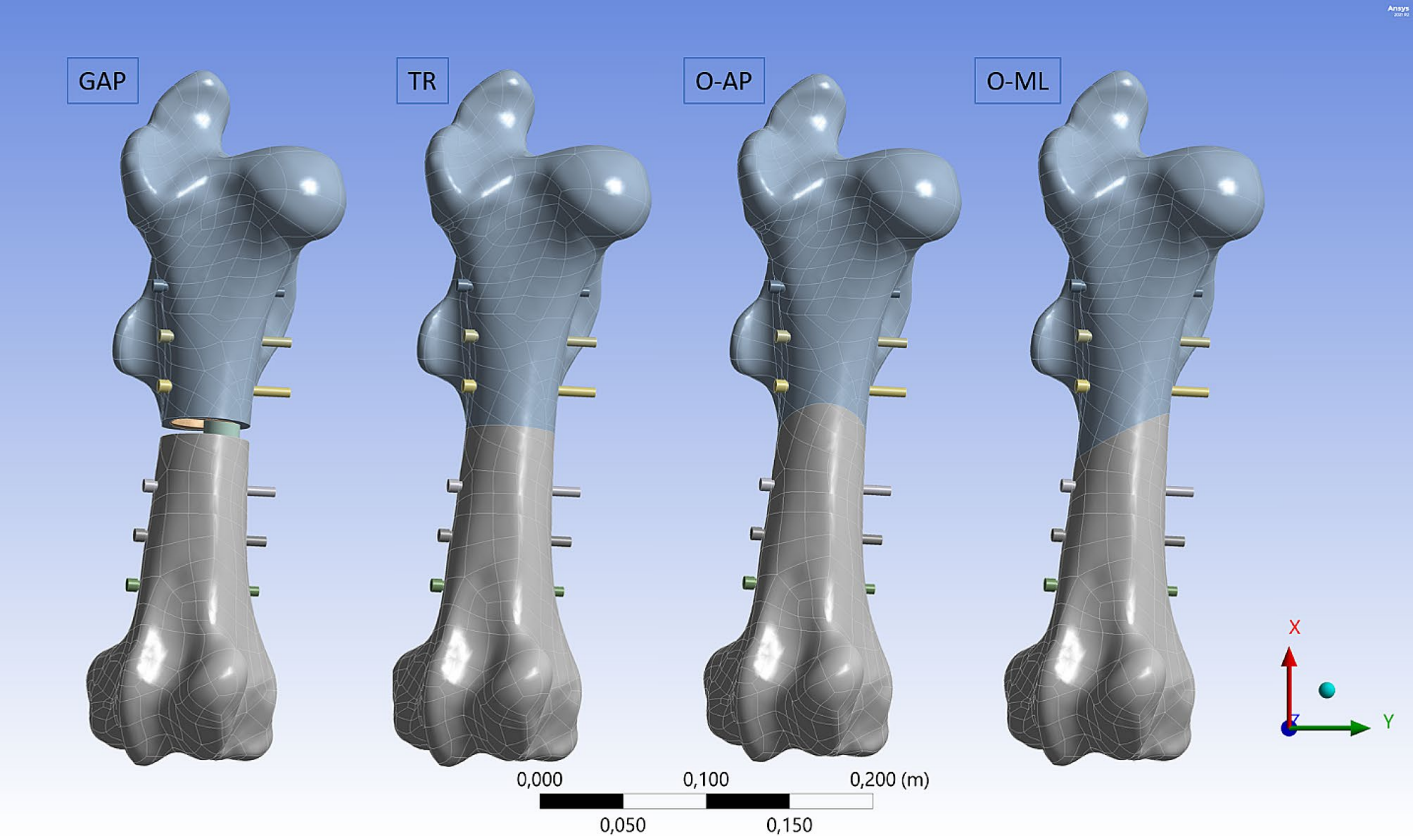
Fracture variations. Four different fracture types were analyzed with FEA. GAP: transverse fracture with a 1 cm gap between the fragments, TR: transverse fracture, O-AP: oblique fracture in anterior-posterior direction, O-ML: oblique fracture in medial-lateral direction. The mesh structure is caused by the reverse engineering algorithms, not the FEA mesh

The geometric parameters of the implant were based on the intramedullary nail for equine osteosynthesis by the company Königsee Implantate GmbH (length: 275 mm, diameter: 22 mm, 6 fixation holes with a diameter of 6 mm). All six holes were filled with screws for fixation (screw diameter: 6 mm). The fixation screws were simplified as cylinders for the numerical evaluation in accordance with the literature [30]. One screw length for all fixation screws was chosen for simplification, which does not resemble the surgery situation, but has no relevance for the results obtained in this study. Additionally, the fracture types were added to the model. Diaphyseal fractures were used because they are common in foals, as there is no record for adults, and their treatment with intramedullary nails provide a satisfactory outcome [1, 11]. Four different types of mid diaphyseal fractures were prepared for evaluation (Fig. 4).

An idealized plain fracture was used, as it has been done for the evaluation of osteosynthesis before [17, 30, 31]. A transverse fracture (TR) is chosen as standard fracture with the highest potential of uncomplicated healing. A transverse fracture with a 1 cm gap between the fragments (GAP) accounts for the often used ’worst case’ scenario in biomechanical *in-vitro* studies [5–10]. An oblique fracture from proximomedial to distolateral (O-ML) direction and an oblique fracture from proximoanterior to distoposterior (O-AP) direction were also incorporated into the model. Fractures of the diaphyseal femur are often accompanied with a torsional overloading and leads to oblique and spiral fractures [32], which is why oblique fracture types were included in this study. For FEA, the four 3D models of bone combined with the implant as well as the sole model of the bone were imported into the numerical simulation software *Ansys* (Version 2021 R2, ANSYS. Software Corporation, Canonsburg, USA). For comparison, also the intact femur without fracture and osteosynthesis was analyzed with FEA. This results in a total of five different simulations.

### Model properties, loading conditions and evaluation

Cortical and cancellous bone as well as the implant material were modeled as linear elastic and homogeneous materials, which is sufficient in most cases of implant stability assessments [15, 17, 30, 31]. The models were meshed with about 500,000 tetrahedral elements and the material parameters are shown in Table 1.

**Table 1:** Parameters for FEA as homogeneous isotropic materials

	Young’s Modulus	Poisson’s Ratio	Ultimate Compressive Strength	Ultimate Tensile Strength
Cortical Bone	17.0 GPa [33]	0.14 [34]	145 MPa [35]	121 MPa [35]
Cancellous Bone	0.28 GPa [36]	0.15^$^	38 MPa [37]	15 MPa [38]
**Titanium Alloy**	**1.1 GPa** [30]	**0.3** [30]	**1661 MPa** [39]	**1115 MPa** [39]

^**$**^ **derived from experimental data not shown here**

**Fig. 5:**
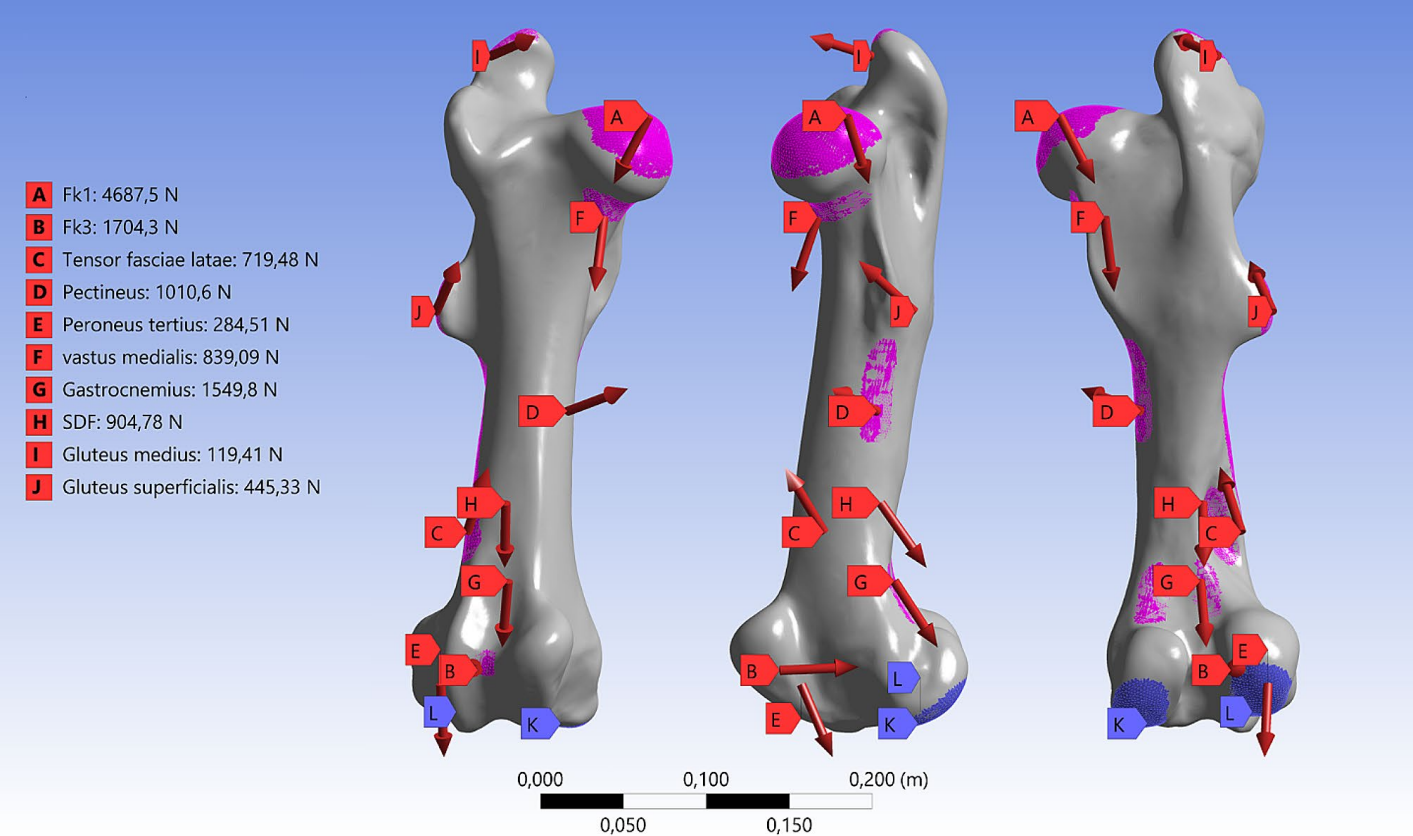
Muscle and contact forces for FEA. Overview on the muscles and contact forces with corresponding areas that were transferred from the musculoskeletal model to the numerical evaluation. The blue areas are the contact areas for the contact forces of the femur with the tibia, which were implemented in the model as fixed support (L) and limited displacement (K)

The convergence study (change of maximum von-Mises stress below 5%) resulted in elements of 4*10^−^³ m, 3*10^−^³ m and 1,5*10^−^³ m for cancellous bone, cortical bone and the implanted material, respectively. The contact areas of the two bone types were defined as bonded. The contact areas between the screws and the bone were defined as bonded, too. Bone-bone contact at the fracture site as well as bone-nail contact was set as frictional contact behavior and their coefficients were defined to 0.46 [31] and 0.37 [40], respectively. Metal-metal contact was described with a coefficient of 0.1 [40]. For the loading conditions in the numerical model, the muscle forces derived from optimization and the defined contact forces were used (Fig. 5). All muscular forces that have an attachment to the femur and exceed 100 N were used for the numerical analysis. 100 N was used as cut off to simplify the simulation as less than about 10 kg of force will not have a major effect on the stress distribution or the stability of the osteosynthesis. The attachment and contact areas on the bone were derived from the literature [14, 23, 24, 41]. The forces were uniformly distributed over the defined areas. In the femoral condyles’ contact region, bearing constraints were established rather than applying direct loads. The medial condyle’s contact area was designated as a fixed support, whereas movement in the proximal-distal and anterior-posterior directions was limited in the contact area of the lateral condyle. This approach was taken to prevent creating a statically overdetermined system, allowing for medial-lateral strain between the condyles. Opting for constraints over direct loading with the femorotibial contact force was a strategic choice to ensure mechanical stability. The resulting forces in the bearing are similar to the contact force from the model. For analysis of the stress distribution in the standing position, static loading was simulated. The first and third principal stresses were used to identify tensile and compressive stress maxima, which is in accordance to other studies on equine biomechanics with FEA [13, 14].

## Results

The resulting forces of the optimization approach for muscles, ligaments and contact forces can be found in Table 2.

**Table 2:** Muscle force result of the optimization approach for the standing horse in the hindlimb

Muscle type	Contraction force in *N*
M. tensor fasciae latae	2158
M. gastrocnemius	1550
**M. pectineus**	**1010**
**M. superficial digital flexor**	**905**
**M. vastus medialis**	**840**
**M. bicep femoris**	**834**
**M. superficial glutea**	**445**
**M. peroneus tertius**	**285**
**M. gluteal medius**	**119**
**M. vastus lateralis**	**66**
**M. adductor muscle**	**63**
**M. gracilis**	**40**
**M. gluteus profundus**	**32**
**M. semimembranosus**	**32**
**M. rectus femoris**	**32**
**M. semitendinosus**	**28**
**M. sartorius**	**24**
**M. extensor digitalis**	**16**
**M. vastus intermedius**	**13**

**Ligament type**	**transfer force in N**
**Lig. patellae intermediale**	**2092**
**Lig. patellae medialis**	**170**
**Lig. patellae laterale**	**0**

*M. tensor fasciae latae, m. pectineus, m. vastus medialis, m. peroneus tertius, m. gastrocnemius, m. superficial digital flexor, m. gluteus medialis* and *m. gluteus superficialis* were used for the FEA analysis. These muscles were connected to the femur and had a resulting muscle force of over 100 N. The resulting contact forces at the joints were 4688 N for the hip joint, 7017 N for femorotibial joint and 1704 N between patella and femur.

**Fig. 6:**
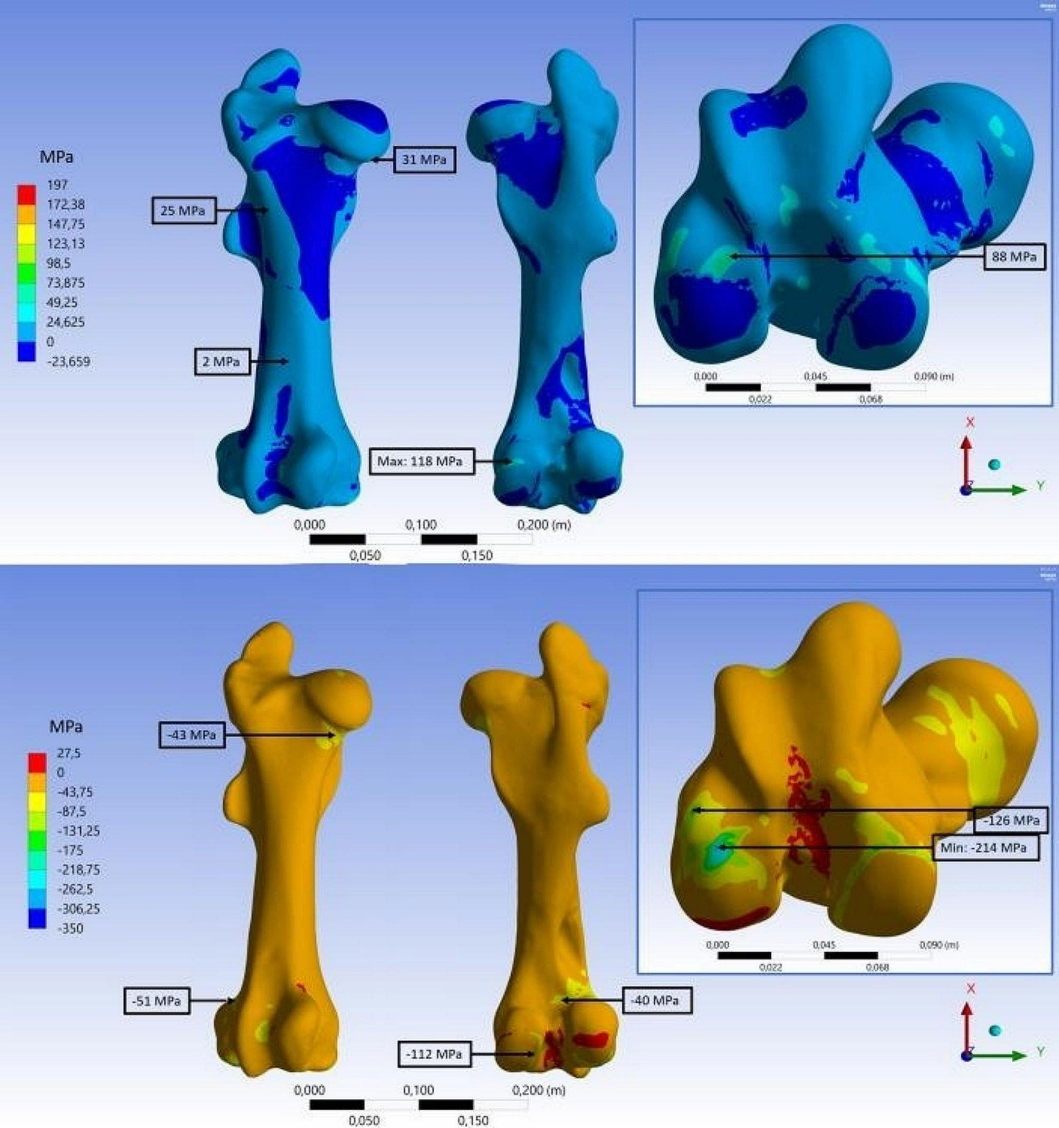
FEA results for tensile and compressive stress on the intact bone. First (top) and third (bottom) principal stress distribution on the intact equine femur in anterior and posterior view. Embedded detail window shows the area of maximum stress at the condyles from distal direction

**Table 3:** Results of the FEA for the intact femur and the different fracture types presented as local maximum tensile and minimal compressive stresses (first and third principal stress)

	*Bone*			*Intramedullary Nail and screws*	
	**Maximum tensile ** **stress in MPa**	**Minimum compressive** stress in MPa		**Maximum tensile** stress in MPa	**Minimum compressive** stress in MPa
**Intact**	118		−214		
**TR**	96		−195	104	−256
**O-ML**	91		−156	157	−274
**O-AP**	96		−140	119	−269
**GAP**	107		−227	197	−348

These forces were transferred into the FEA. The average calculation time was 2 h and 8 min for the FEA. The stress results for intact bone as well as for all fracture types are given in Table 3. For the intact bone, the maximum tensile and compressive stresses (118 MPa and − 214 MPa) were both located in the cortical bone at the lateral condyle of the femur (Fig. 6). Also increased tensile stress is located at the lateral condyle, at the anterior edge of the condyle surface (88 MPa), and at the edge of the femoral head (31 MPa). Increased compressive stress areas are found proximally to the condyles (-40 MPa), between condyles and fossa supracondylaris, and on the medial metaphysis located distally of the head of the femur (-43 MPa).

**Fig. 7:**
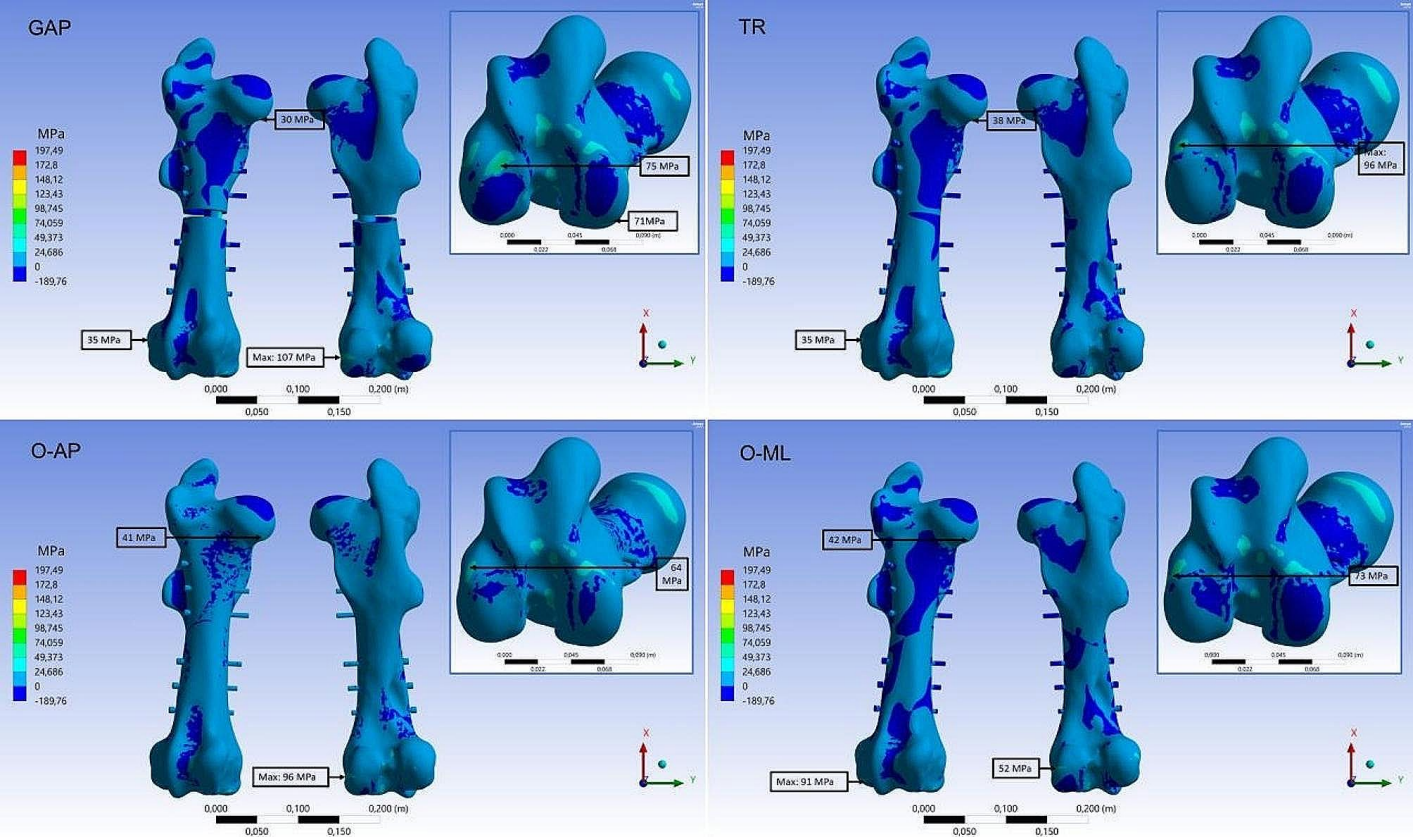
FEA results for tensile stress. First principal stress distribution on the cortical and cancellous bone for the four fracture types (GAP, TR, O-AP and O-ML). Embedded detail window shows the area at the condyles from distal direction

**Fig. 8:**
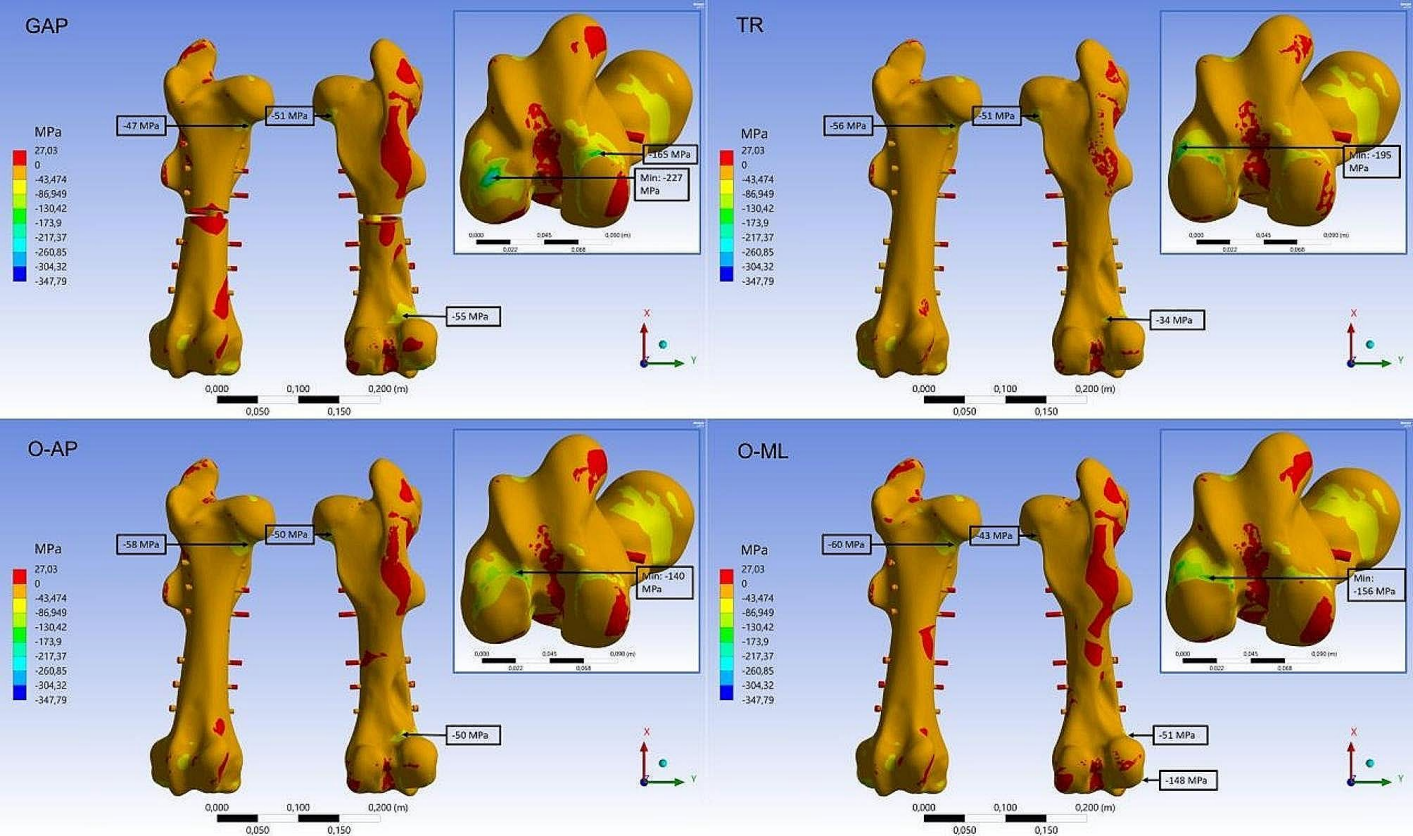
FEA results for compressive stress. Third principal stress distribution on the cortical and cancellous bone for the four fracture types (GAP, TR, OL-AP and O-ML). Embedded detail window shows the area at the condyles from distal direction

The stress distribution on the bone material was altered in the different fracture types (Figs. 7 and 8). Especially in the diaphysis, the compressive stresses are reduced due to force transfer caused by the implant. But the peak values in the cortical bone are in a similar range. The compressive stress maximum is located on the cortical bone at the lateral condyle for all fracture types (O-ML: -156 MPa, O-AP: -140 MPa, GAP: -227 MPa, TR: 195 MPa). For the tensile stresses, the maximum values are located at the lateral condyle for the TR fracture (96 MPa) and O-ML fracture (91 MPa), similar to the intact bone. For O-AP fracture and GAP fracture, the maximum tensile stress is found in the cortical bone at the transition of the medial condyle to medial epicondyle (O-AP: 96 MPa and GAP: 107 MPa).

**Fig. 9:**
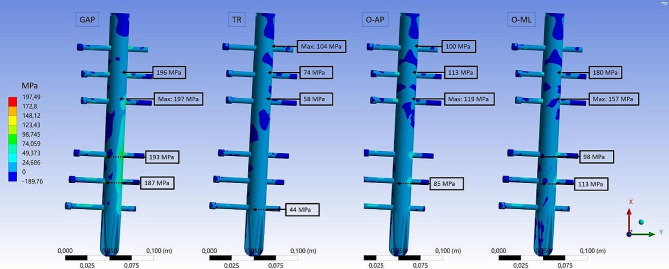
FEA results for tensile stress. First principal stress distribution on the intramedullary nail and the screws for the four fracture types (GAP, TR, O-AP and O-ML).

**Fig. 10:**
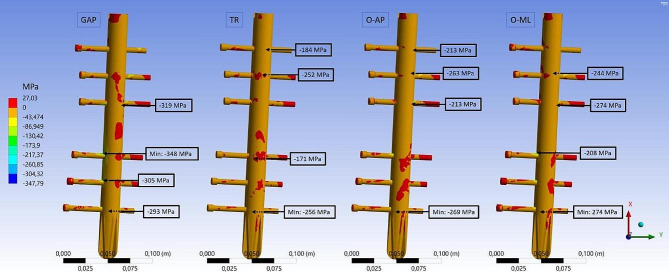
FEA results for compressive stress. Third principal stress distribution on the intramedullary nail and the screws for the four fracture types (GAP, TR, O-AP and O-ML).

A difference between the stress distribution of the GAP fracture and the other fracture types was most pronounced for the implanted parts. The comparison can be observed in Figs. 9 and 10. Maximum values for the stresses are located all on the screws. For the TR fracture, the maximum compressive stress (-256 MPa) and the maximum tensile stress (104 MPa) is located on the first (most proximal, see Fig. 3) and last (most distal) fixation screw at the contact area with the intramedullary nail. For both oblique fractures the maximum compressive stress was at the same site at the most distal screw (OML: -274 MPa, O-AP: -269 MPa). For the tensile stress, the oblique fractures also had the maximum value at the third screw at the contact area with the intramedullary nail (O-ML: 157 MPa, O-AP: 119 MPa). GAP fracture showed a local maximum tensile stress of 197 MPa at the third screw and also relatively high stress for the second, fourth and fifth screw. Compressive stresses (GAP: -348 MPa) maxed on the fourth screw on the lateral site close to the edge of the nail.

High compressive stresses were found at the point where the screw contacts the intramedullary nail more centrally. In contrast, tensile stress was observed to increase near the medial or lateral sides from that point on the screw. Overall, stress levels were highest for the GAP fracture and lowest for the TR fracture.

## Discussion

The musculoskeletal model described in this work provides information about the distribution of the muscular loading in the upper part of the hindlimb during standing, which is lacking in the literature so far. The forces and their distributions obtained are based on several assumptions and simplifications such as reducing the attachments to single points and neglecting geometry changes caused by muscle contraction. Therefore, the result corresponds to an estimation of the real conditions. To check the physiological relevance of the results, the forces can be compared with muscle activity measuring by EMG (electromyography). Schuurman et al. performed a study, in which the activity of muscles attached to the patella in different standing situations were analyzed [29]. They found mainly activity in the *m. vastus medialis*, which also provides distinctive force in our model. But Schuurmann et al. were especially focused on the locking mechanism of the patella in the standing horse. This mechanism is not incorporated in the model described here, which is why the muscles attached to the patella compensate this patellar fixation mechanism. This might be the reason, why we obtained high forces on the *m. biceps femoris* and *m. tensor fascia latae*, despite no significant EMG signal was observed by Schuurmann et al. Furthermore, Frazer et al. used 1000 N as assumption for the *m. quadriceps* force for FEA of the stifle joint [13, 14], which is in agreement with our findings of 951 N for this muscle combination with the *m. vastus medialis* as most contributing component.

The FEA of the intact femur provided insights on the stress distribution of the femur while standing. In general, for the intact femur, a homogeneous stress distribution with increased compressive stress areas is observed. High punctual stresses (about 150% of compressive strength of cortical bone) were present in the condyle area due to the contact conditions between the femur and the tibia, which are defined as fixed support and allow only limited deformation at the surface. This creates unphysiological stress concentrations in the surrounding bone tissue caused by the abrupt transition of the deformation condition. In reality the meniscus and the cartilage of the joint provide for an optimal distribution of the stresses in the joint. As a consequence, the mean maximum stresses can probably be estimated to be slightly lower than those calculated here for the bone. Nevertheless, the FEA provides insights on the loading distribution in the femur while standing, which have not been described in the literature before. The results confirm that the cranial-lateral side of the femur is the tension side. This corresponds with the fact that plate fixation is mainly performed on this side for osteosyntheses in foals [10, 32]. However, stress state is complex and the side-specific differences in the stresses are only slightly pronounced (Fig. 6). From the stress distribution of the intact femur, areas of high stress could be identified that are a potential risk for the osteosynthesis of the bone. In unfavorable configurations, a disruption in the complex bone structure with e.g. a fixation screw can make it more susceptible to mechanical failure [42]. The cortical bone in the caudal area located proximally to the condyles is not altered by the osteosynthesis material. However, for the area located distally to the femoral head with increased compressive stress, two fixation screws penetrate the cortical bone in this area of the fracture models.

As a consequence, the influence of the internal fixation on the stress distribution of a fractured bone was also numerically analyzed. The FEA of the different fracture types showed no distinctive increase of stress in distal direction from the femoral head at the screw penetration sites. A comparison of the stress distribution in distal direction from the femoral head can be made using Figs. 6 and 8.

Similar to the intact bone, the FEA of the fracture types resulted in high stresses at the distal contact force which was modeled as fixed support for the lateral condyle. Some of these values are above the ultimate strength level of cortical bone (Table 1), which means that a theoretical risk of failure is present at these locations. But it is unlikely that this local stress rise will lead to a failure of the bone. It may be the result of unfavorable contact conditions for the fixed supports. Elastic support instead of a fixed support at this area might reduce the local stress maxima and resemble better the real environment with the stress distributing properties of the meniscus and the cartilage as it was mentioned for the intact bone. For a further evaluation an experimental setup with simplified loading condition would be helpful. But for the investigation of the osteosynthesis material this should not alter the insights on the stresses for the implant material.

For the different fracture types, increased stresses were mainly concentrated on the implanted material, especially the fixation screws. This can be observed in Figs. 9 and 10. The screws showed increased stress in contact areas with the intramedullary nail. This implies that the highest risk of osteosynthesis failure is associated with the screws. Even though, the stresses obtained in this study were below the ultimate strength values of the implant material (Table 1). Nevertheless, it may happen during surgery that not all fixation holes in the intramedullary nail can be successfully filled. In this case, the load must be transferred by a reduced number of screws. This increases the potential risk of overloading. And when dynamic loads, such as uncontrolled movement during recovery from anesthesia, and non-optimal implant positioning are also considered, the risk increases even further. Therefore, as many of the six present implant holes as possible should be used for fixation during surgery. A detailed evaluation of the stress distribution introduced by a reduced number of screws can be analyzed in future studies.

The peak stress values of fractured femora and the native femur shown in Table 3 were relatively comparable. This indicates that the main part of the load is sufficiently transferred through the fracture via the intramedullary nail. Also, almost no relative displacement of the fragments is shown at the fracture site. Consequently, only few transverse forces act on the diaphysis in this loading situation. O-ML, O-AP and GAP showed a similar tensile stress distribution with the maximum stresses on the third fixation screw. But the maximum stress values increase from O-AP over O-ML to the GAP fracture situation. The fracture angle of O-AP seems to better preserve a load transfer from proximal to distal direction through the bone as compared to O-ML. For GAP, the load has to be transferred solely by the implant and the screws, which is why the highest peak stresses are expected for this fracture configuration. Interestingly, increased tensile stresses are observable on the medial side of the intramedullary nail, which would be expected on the lateral side as it is for the bone. The forces generate a lateral displacement of the proximal fragment, which explains the stress distribution on the implant. At the same time, however, a medial bending is initiated via the femoral head, which places the cranio-lateral bone surface under tensile stress. Additionally, for this fracture type, the highest local values of compressive (-348 MPa) and tensile stress (197 MPa) are on the third and fourth screw at the contact with the intramedullary nail, which gives these fracture adjacent screws the highest risk of failure. These stresses are still below the Ti6Al4V yield strength of -1074 MPa and 982 MPa, respectively [39]. Thus, no plastic deformation is expected and a safety factor of 3.1 and 5.0 can be calculated.

There are several limitations to the present study that need to be addressed. For the musculoskeletal model, it is important to note that antagonistic co-contraction cannot be properly considered with the optimization method described in this manuscript. The moments caused by antagonistic and agonistic muscles are canceled out during the optimization process. But for the standing horse, these co-contractions may be important for joint stabilization [43]. The implementation of a minimum activity for all muscles reduces the resulting divergence, but only to a small extent. Consequently, this optimization approach tends to underestimate the contact forces in the joints [43], which must be taken into account in the design of the implant with an appropriate safety factor. Furthermore, using a Haflinger bone is a limiting factor because these are smaller horses with a stocky build. But even though this configuration is not ideal to represent the entity of all horses, it provides insights on the stress distribution of the bone and potential weak spots of the osteosynthesis. In addition, the approach presented here only analyzes the forces while standing. This is a static assumption that is justified for the initial rehabilitation period after surgery. For an extended analysis, it would also be interesting to consider the dynamically induced loads. The use of a multi-body simulation would be suitable for this. Motion tracking and gait analysis can be used to determine the forces acting on the femur using inverse dynamics [44, 45]. These determined forces can then be transferred to a corresponding FEA in experimental studies [46].

For the numerical analysis, the main limitation is that it is not possible to validate the model because nothing is known about the stress distribution of the femur in horses while standing. As mentioned above, the stress distribution in the proximal equine femur following a cyst lesion was investigated in an FEA by Frazer et al., but rough assumptions were made for the loading [13, 14]. Moreover, stress distribution is only shown in a small region around the lesion, where the highest stresses were present, which makes these studies insufficient for validation. Furthermore, the numerical loading in the present study does not include the stability that is given to the bone by the ambient pressure caused by the surrounding muscle tissue. This can affect the stress distribution in the bone and enhance the osteosynthesis stability especially considering contracting muscles. Furthermore, it has to be stated that a numerical analysis is in most cases a tool for approximation of the relative behavior in different scenarios rather than an exact prediction. This is especially true with biological materials. The homogeneous isotropic material properties we used in this study are a simplification of the very complex material behavior of cancellous and cortical bone. These simplifications are often necessary for a sufficient analysis of heterogeneous systems. Based on these conditions, all osteosynthesis situations investigated here provided mechanically stable fixation without areas prone to failure, apart from the small bone regions discussed above. However, to further increase the safety of intramedullary fixation for the possibility of asymmetric, dynamic loading or a reduced number of sufficiently applied fixation screws, screws with a bigger diameter could be used. This is especially true at the intramedullary nail holes adjacent to the fracture.

## Conclusions

The work shown here introduced a musculoskeletal model of the loading situation for the equine hind limb while standing, which was lacking in the literature so far. The model was used to analyze stress distribution of the intact femur and four different fracture situations after osteosynthesis with an intramedullary nail. The fixation screws were found to obtain the highest stresses especially for the GAP fracture. The entire osteosynthesis is shown in the numerical analysis to be stable for all analyzed fracture types and designed with a sufficiently high safety factor for the implant material. The model as well as the findings from this study provide a rapid mechanical evaluation setup to further optimize equine femur osteosynthesis, in which primary stability is extremely important.

### Electronic supplementary material

Below is the link to the electronic supplementary material.

Supplementary Material 1

Supplementary Material 2

Supplementary Material 3

## Data Availability

The datasets generated during and/or analysed during the current study are available from the corresponding author on reasonable request.

## Data availability

The datasets generated during and/or analysed during the current study are available from the corresponding author on reasonable request.
